# WAITING TO BENEFIT: AGE-BASED DISABILITY REGULATIONS AND PATHWAYS TO SUPPLEMENTAL SECURITY INCOME IN LATER LIFE

**DOI:** 10.1093/geroni/igae098.2172

**Published:** 2024-12-31

**Authors:** Callie Freitag

**Affiliations:** University of Wisconsin-Madison, Madison, Wisconsin, United States

## Abstract

Supplemental Security Income (SSI) is an important resource for low-income older adults and people with disabilities, but it is notoriously difficult to be deemed eligible. Older SSI applicants may have an easier time getting approved. Starting at age 45, the Social Security Administration (SSA) relaxes the disability requirements for certain groups every five years until they are eliminated at age 65. If the age-based rules prevent otherwise-eligible people from receiving SSI benefits, then the consequences for this economically vulnerable group could be particularly dire. This paper has two aims. First, I estimate the SSI take-up rate by age and test whether discontinuities in SSI take-up exist when SSA disability determination rules are relaxed (at ages 45, 50, 55, and 60) and eliminated (at age 65). Second, I identify patterns of employment, homelessness, and public assistance use among SSI recipients in the five years prior to SSI take-up and examine how these patterns vary by age. I conduct these analyses with administrative microdata from multiple agencies in Washington state spanning from 2010 through 2017. I find strong evidence of discontinuous increases in SSI take-up when the disability rules are relaxed at age 55 and eliminated at age 65, suggesting the program rules may act as gatekeepers for otherwise eligible applicants. Adults face significant financial hardship in the five years prior to receiving SSI. People who took up SSI between the ages of 40 and 59 were more likely to have been homeless than employed prior to receiving SSI.

